# Developing a Polypropylene Fabric, Silica Fume, and Redispersible Emulsion Powder Cementitious Composite for Dynamic Water Environment

**DOI:** 10.3390/polym11010047

**Published:** 2018-12-30

**Authors:** Jinquan Liu, Xiaofei Li, Pooya Saffari, Qichao Liang, Ling Li, Weizhong Chen

**Affiliations:** 1 School of Civil Engineering and Architecture, East China Jiaotong University, Nanchang 330029, China; jinquanliu99@163.com; 2 Quanzhou Institute of Equipment Manufacturing, Haixi Institutes, Chinese Academy of Sciences, Quanzhou 362000, China; pooyasaffari@fjirsm.ac.cn (P.S.); qichaoliang1984@163.com (Q.L.); 3 State Key Laboratory of Geomechanics and Geotechnical Engineering, Institute of Rock and Soil Mechanics, Chinese Academy of Sciences, Wuhan 430071, China; 4 CCCC Railway Consultants Group Co., Ltd, Beijing 100088, China; lzjtulixiaofei@163.com; 5 Nanchang Institute of Technology, Nanchang 330029, China; qwas6248178@163.com

**Keywords:** groundwater control, dynamic-water environment, polypropylene fiber, silica fume, redispersible emulsion powder, composite slurry

## Abstract

In the dynamic water environment, grouting requires a material with higher strength and anti-washout performance to prevent groundwater inrush. This study aims to develop a dynamic water slurry by mixing polypropylene fiber (PP fiber), silica fume (SF) and the polymer material of redispersible emulsion powder (REP) to the Portland cement. Towards this aim, a series of tests, including strength, gel time, bleeding rate, fluidity, and anti-washout, were conducted to evaluate the effects of SF, PP fiber, and REP on the slurry properties. The test results show that: (1) SF displays significant effects on strength, gel time, fluidity, and bleeding rate of cement slurry. Differently, PP fiber mainly affects the stress–strain behavior of the slurry and can improve the ductility significantly. (2) By mixing SF and fiber simultaneously, the slurry strength can increase by about 30%, and its strain can extend by more than 70%. Meanwhile, the composite slurry possesses great anti-washout properties at a low flow velocity (*v* ≤ 0.4 m/s), and the grouting retention rate (*GRR*) can reach up to 98.7%. However, the *GRR* decreases to a maximum value of 31.3% when *v* = 0.6 m/s. (3) By mixing the REP into the fiber-SF composite slurry, the *GRR* can further increase, reaching more than 60% even when *v* = 0.6 m/s. As a result, the developed fiber-SF cementitious composite slurry, which when mixed with REP, presents a favorable performance in the dynamic water environment.

## 1. Introduction

Tunnel and underground engineering constructing in unfavorable geological zones, i.e., karst, faults, and weathered zone, often face the serious risk of groundwater, especially fast-moving water [1,2,3,4,5]. In recent years, more than 100 groundwater inrush disasters have been observed across the world [6,7], presenting a significant challenge of groundwater control to the engineers. Grouting, as the common treatment measure, has far from the desired effect in the dynamic water environment due to the water erosion [8]. The grout in this condition would be diluted and scoured, which decreases the effect of water plugging and the ground reinforcement [9]. Therefore, the grouting material with a favorable performance of strength and anti-washout are strongly demanded in the dynamic water environment [10].

Presently, the widely used grouting materials for groundwater control mainly include cement slurry, cement-sodium silicate binary slurry, and chemical slurry. The application of these materials in the dynamic water condition face the following problems. First, the cement slurry in the abundant water with velocity is prone to be diluted and scoured due to the long gel time (higher than 3 h), which would lead to the rapid decrease of the grouting effect [11]. Numerous experimental studies demonstrated that the grouting retention rate (*GRR*) of cement slurry in this condition is less than 40% [12]. In addition, the water syneresis rate is relatively high, both resulting in a lower later slurry strength (the residual compressive strength of a sample cast in the underwater environment is only about 65% of that in the air [13]). Second, the cement-sodium silicate binary slurry possesses a short gel time (20–60 s in general, considering the ratio of cement and sodium silicate) [14]. Simultaneously, this binary slurry possesses favorable anti-washout properties for a lower water velocity (≤0.4 m/s), which in this case, the *GRR* can reach up to 86.2% [15]. Unfortunately, the admixture of sodium silicate causes a serious effect on the cement strength, which may decrease more than 40% of the cement strength [16]. Meanwhile, the volume of slurry in the later stage may retract and the strength may reduce, which can cause cracks and decrease the reinforcement effect [17]. Furthermore, the *GRR* of slurry in high flow water (>0.4 m/s) is less than 20%, indicating that this slurry is quite insufficient for the anti-washout. Third, the chemical slurries, i.e., polyurethane slurry and Malisan slurry, possess potential performance of anti-washout. In chemical slurries, the *GRR* can reach up to 90% even in a high flow water environment (*v* > 0.4 m/s), and thus they are widely used in many emergency projects. However, due to the expansibility behavior in most of them, the gel time is extremely short (smaller than 40 s), and the grouting pressure rises extremely fast. This is prone to result in the pipe blockage and large deformation in the ground [18,19]. Furthermore, the toxicity and expansive cost of these materials may pollute the groundwater environment and further restrict the wide engineering applications [11,20,21].

Therefore, it is necessary to develop a dynamic-water slurry that possesses favorable performances, such as strength and anti-washout characteristics, to prevent the groundwater flow disasters. Han et al. [22] and Usman et al. [23] declared that the fiber incorporated into the cement slurry can improve the tensile strength and deformability. Specifically, the addition of polyolefin fibers can increase flexural strength by up to 13% and reduce the growth or propagation of cracks by up to 70% compared to control specimens. Furthermore, numerous experimental studies demonstrated that the compressive strength can be improved significantly by incorporating silica fume (SF) into the cement slurry [24,25,26]. Those studies indicate that the fiber and SF are favorable materials for modifying the strength and deformability performance of cement. However, these studies did not involve in the water environment, and neglected the anti-washout performance after adding the SF and fiber into cement. In terms of anti-washout behavior, Jiang et al. [27] and Han et al. [28] declared that the admixtures of polymer materials, i.e., cellulose-based methylcellulose and polythene-based polyacrylamide, can ameliorate the anti-washout characteristic, greatly reduce bleeding, and significantly enhance the resistance of concrete to water dilution and segregation. However, these admixtures may result in significant air-entrainment, water demand, and strength loss, and the effects increase with the increased admixtures dosage. Fortunately, these studies indicated that single or multiple polymer admixture materials with appropriate proportions may obtain excellent underwater and anti-washout performance, which may be an important direction to optimize the cement behavior in dynamic water. 

In this paper, on the basis of the cement slurry, materials of polypropylene fiber (PP fiber), SF, and polymer material of redispersible emulsion powder (REP) were considered in the improvement of the cement behaviors. The strength, gel time, bleeding rate, and anti-washout characteristics of the composite slurry were systematically investigated, and a new green grouting material that can guarantee the later strength and effectively resist the erosion of dynamic water was developed.

## 2. Materials and Testing Design

### 2.1. Raw Materials

The Portland cement used in this study (type P.C.32.5 in accordance with the relevant Chinese standard) was from Huaxin Cement Plant factory (Huangshi, China). In addition, the SF (with a fineness of 1.6 μm and a specific surface area of at least 15 m^2^/g) and PP fiber were provided from Zibo Hanye Refractories company (Zibo, China) and Shanghai Chenqi Chemical Technology company (Shanghai, China), respectively. The REP was supplied from Heyday Mobil company (Shanghai, China), and the chemical composition was mainly ethylene vinyl acetate (EVA) [29,30,31]. The performance index of cement, SF, PP fiber, and REP, which are used for this study, are presented in Table 1, Table 2 and Table 3.

### 2.2. Testing Procedure and Testing Design

A series of tests including strength, gel time, and bleeding ratio tests were carried out to study the influence of SF and PP fiber on the properties of cement at first. Then, strength tests with different proportions of SF and PP fiber mixing into the cement were conducted to investigate the optimal proportion for the cementitious composite strength. Based on the optimal proportion, the gel time, fluidity, bleeding rate, and anti-washout of the cementitious composite were evaluated. At last, the REP was used to improve the anti-washout properties.

Tests considered the factors of the water-cement ratio (w/c) of Portland cement and mixing amount of SF and PP fiber. In addition, the REP was considered as an admixture to improve the anti-washout performance. To prepare the samples, dry powders of cement, PP fiber, SF, and REP were put into the mixer and mixed for 3 min considering the fiber was difficult to disperse in water. After that, the water was put into the mixer and mixed for 4 min. Afterward, the fresh mixtures were cast into steal molds and compacted via a standard vibrating table. The specimens were demolded after 24 h and cured under standard conditions (20 ± 2 °C, RH > 90%) for designated ages of 7 days before testing.

**Figure 1:**
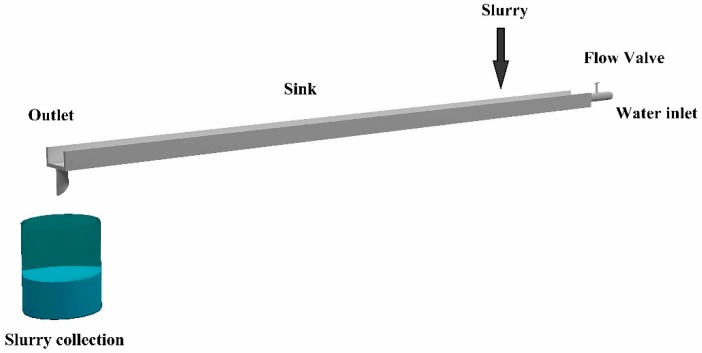
Sketch of anti-washout property test apparatus.

The details of the test method and scheme were as follows:

(1) The w/c ratio with 0.8, 1.0, and 1.2 by weight; SF proportion with 5%, 10%, and 15% of cement quality; and PP fiber proportion with 0.1%, 0.3%, and 0.5% of cement volume were selected to carry out various tests.
(2) Gel times (initial and final) were investigated by conducting Vicat needle tests according to the standard GB/T1346-2011 of China. Specimens for Vicat needle tests were formed by placing the needle in the suspension, immediately after preparation.
(3) Fluidity was evaluated using a fluidity test apparatus, which consisted of a mixer, a truncated cone model, and a glass plate according to the standard GB/T8077-2000 of China. First, the truncated cone model was placed in the center of the glass plate. Then, the prepared fresh mixtures were put into a truncated cone model immediately. After that, truncated cone model was lifted and the diameter of the mixture flow in the glass plate was measured for 30 s. The average value of the maximum diameter at the mutual vertical directions was defined as the fluidity.
(4) Bleeding rate was investigated by conducting sedimentation tests. Immediately after fresh mixtures preparation, 1000 mL of the mixtures were placed in a capacity cylinder (namely graduated beaker) and the volume of sediment was recorded as a function of time. Measurements were taken at predetermined time intervals for a period of up to 24 h to ensure that sedimentation had been completed. The bleeding rate is a function of time and is defined as the volume of bleed water above the slurry, Δ*V*, expressed as a percentage of the total volume of the initial slurry, *V_0_*. The value of Δ*V*/*V_0_* (after the end of sedimentation) is defined as the bleed capacity of the mixtures.
(5) After 7 days standard curing, specimens with the size of 40 × 40 × 160 mm^3^ were prepared for the static mechanical strength tests. Flexural strength and compressive strength were measured using rock and concrete mechanics test system (RMT-201) according to the standard GB/T50081 of China. First, the three-point bending test was performed to investigate flexural strength. Six samples were tested for each mix. The average value was served as the final flexural strength after removing the maximum and minimum values in the six samples. After the bending test, the broken two parts were used to conduct the compressive test. Three samples were tested for each mix. The average value was obtained as the final compressive strength.
(6) The anti-washout properties were evaluated by a self-design testing apparatus, as shown in Figure 1. The apparatus was composed of a sink, flow valve, and slurry collection equipment. The length, width, and height of the sink was 200, 10, and 5 cm, respectively. The flow valve was used to adjust the flow velocity. Three water velocities, namely 0.2, 0.4, and 0.6 m/s, were considered in the tests. Furthermore, the procedure of the anti-washout test was illustrated as follows. First, the slurry with mass *M*_0_ (380 g in all the tests) was poured into the sink under the designed dynamic-water velocity. When the water in outlet changed to clean, the water inlet was closed, the residual slurry in the sink was collected and the mass *M*_1_ was recorded. Then, the anti-washout properties were evaluated using the index of grout retention ratio (*GRR*), which was defined by the ratio of *M*_1_/*M*_0_.

## 3. Result and Analysis

### 3.1. Influence of PP Fiber and Silica Fume on Cement Slurry

On the basis of Portland cement with varying w/c (0.8, 1.0, 1.2), a comprehensive test method was used to evaluate the performance modification of composite material by mixing different amounts of PP fiber (0.1%, 0.3%, 0.5%, respectively), and SF (5%, 10%, 15%, respectively). The test results are shown in Table 4. Table 5 presents the statistical analysis results of compressive strength (other results are not listed considering the analysis results are similar), which indicates that the factors except PP fiber possess statistically significant influences on the slurry performances, i.e., strength and bleeding rate. The detailed results analysis is listed in the following.

#### 3.1.1. Strength Properties of Composite Material

(a) Strength optimal proportion

**Figure 2:**
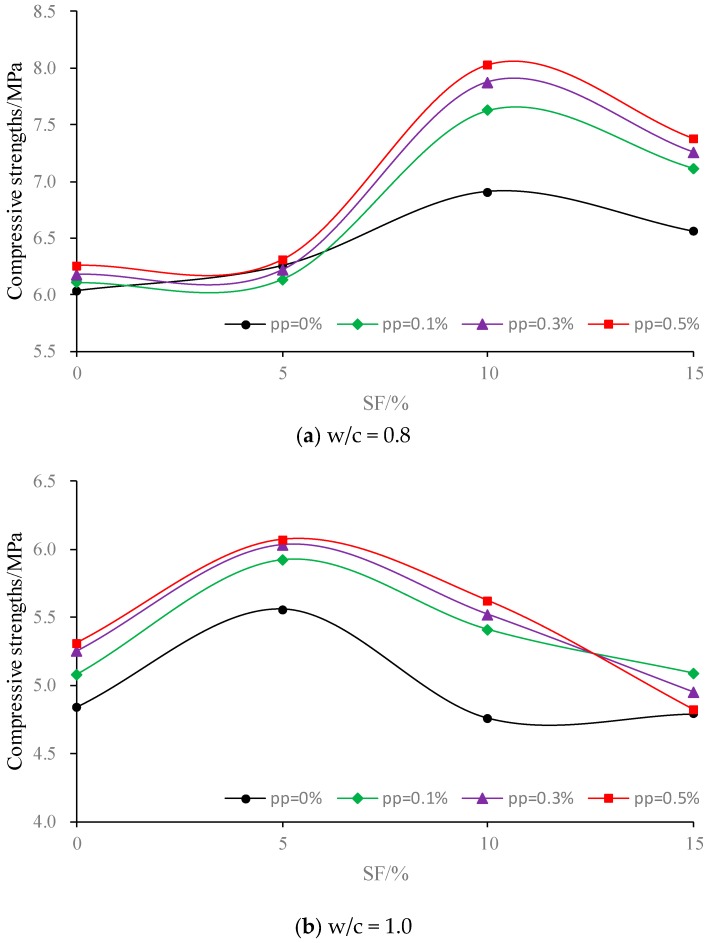

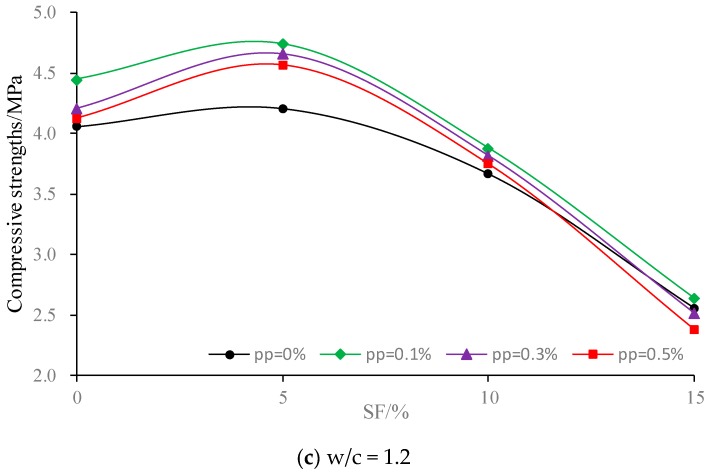
Compressive strength of composite fiber-silica fume cement under different w/c.

Figure 2 presents the compressive strength and flexural strength of composite materials under different mixing amount of PP fiber and SF. From Table 4 and Figure 2, the following results can be obtained:

(1) Compared with the plain cement mixture, the maximum increase ratios of flexural strength and compressive strength after mixing the PP fiber individually reached 11.0% and 9.7% (at w/c = 1.0 and pp = 0.5). Meanwhile, those ratios after mixing SF individually increased to 13.2% and 14.4% (at w/c = 0.8 and SF = 10%), which are shown in Table 4. This indicates that SF had a greater influence than PP fiber on slurry strength.
(2) As shown in Figure 2, the strength increased first and then decreased with the increase of SF, indicating a proper mixing amount existed for the modification of strength. Otherwise, the strength will in turn decrease with the further increase of SF, particularly, the flexural strength, which decreased to 0.46 MPa when SF = 15% at w/c = 1.2. This may have been due to the fact that the proper mixing amount of SF could fill the pore of cement powder, and then increase the strength. However, with the significant increase of SF, the number of cement particles will in turn decrease and weaken the hydration reaction, which will lead to the decrease of strength.
(3) As shown in Figure 2, it can be observed that w/c possessed a negative influence on the strength. As the w/c increased, the strength decreased significantly. While the PP fiber possessed a positive influence on the strength at a low w/c (i.e., 0.8), this influence significantly reduced at a high w/c, or even transferred into the side effect with the increasing fiber amount. This may due to the fact that the interfacial bonding between the fiber and cement can increase the slurry strength at a low w/c. However, the porosity of slurry will increase with the increase of w/c, and the high amount of fiber will decrease the interfacial bonding and decrease the strength. Similarly, at the low w/c (i.e., 0.8), the suitable SF content was beneficial for the strength growth, while at a high w/c (i.e., 1.2), the strength decreased with the increase of SF, and the lower SF content was more beneficial for strength growth.
(4) Table 4 indicates that there is an optimal proportion of SF and PP fiber content to maximize the strength for various w/c ratios. In addition, when w/c = 0.8, the optimum proportion (OP) of SF and PP fiber was 10% and 0.5%, respectively. Furthermore, when w/c = 1.0, the OP was 5% and 0.5%, respectively, while when w/c = 1.2, the OP was 5% and 0.1%, respectively.

(b) Comparison with Portland cement strength

Table 6 presents the strength comparison results of the plain cement mixture and fiber-silica fume composite slurry at the optimum proportion. It can be observed that the compressive and flexural strength of the composite slurry were significantly higher than that of plain cement mix. The strength growth rate of cement with the low w/c was larger than the high w/c. When w/c = 0.8 and 1.0, the compressive and flexural strengths were both increased by about 30%. While when w/c = 1.2, the compressive and flexural strengths were increased by more than 10%.

(c) Stress-strain curve

**Figure 3:**
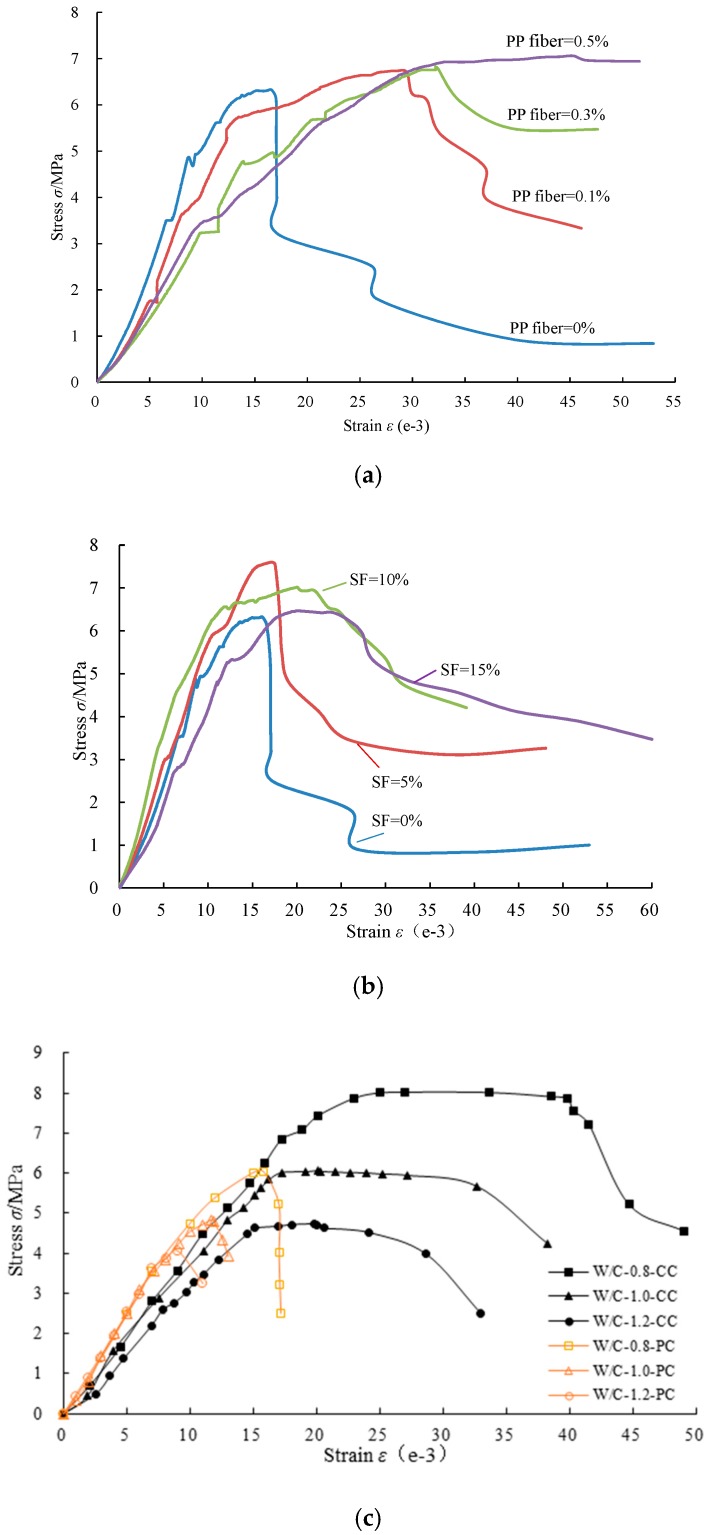
Stress-strain curve of cementitious mixtures: (**a**) Fiber cementitious mixtures at w/c= and curing age = 7 days; (**b**) SF cementitious mixtures at w/c = 0.8 and curing age = 7 days; (**c**) Fiber-SF cementitious composite (CC) and plain portland cement (PC) at curing age = 7 days.

Figure 3 presents the stress-strain characteristics of fiber and silica fume cementitious mixtures. As shown in Figure 3a, the PP fiber displayed a serious influence on the stress-strain characteristics and failure model. For the plain cement, the strain at peak stress was 16.13 × 10^−3^. When PP = 0.1%, 0.3%, and 0.5%, the strain increased 73.2%, 99.6%, and 144.1%, to 27.93 × 10^−3^, 32.20 × 10^−3^, and 39.37 × 10^−3^, respectively, indicating that the fiber cement could hinder the crack propagation and ameliorate the brittle failure model to ductile failure, especially when pp = 0.5%. However, due to the incorporation of the PP fiber with a lower elastic modulus, the stiffness seemed to decrease with PP fiber increase. Figure 3b indicates that the strain at peak stress increased with the increase of SF. However, the SF mixing into cement could not change the brittle failure model.

**Figure 4:**
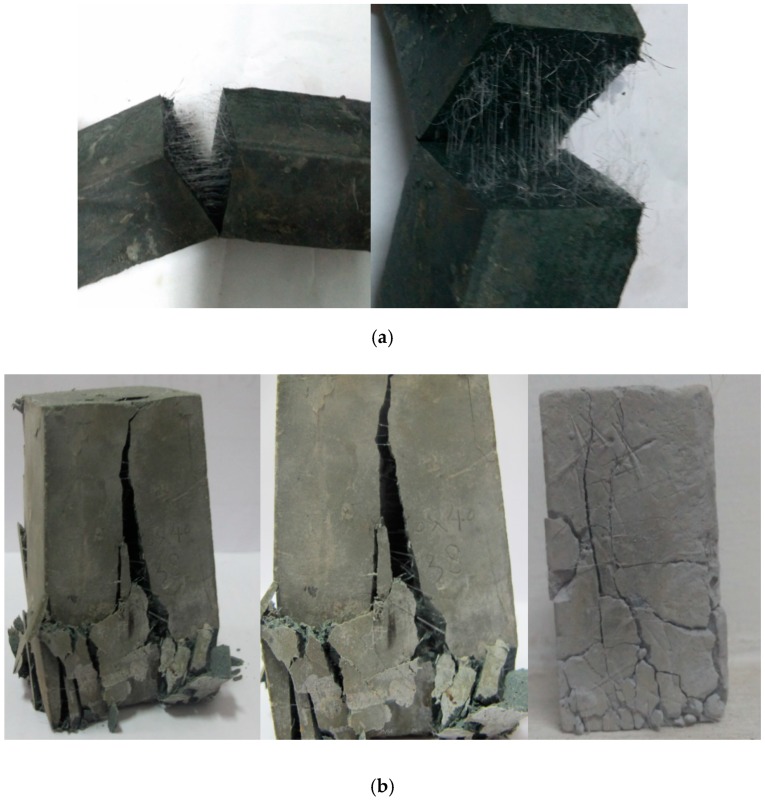
Failure type of specimens subjected to strength tests: (**a**) Flexural strength specimen; (**b**) Compressive strength specimen.

Figure 3c shows the stress-strain curve of plain cement and fiber-SF cementitious composite slurry at the optimal proportion. For the plain cement, the residual strength remains low and the failure model performs brittle fracture. With the decrease of w/c, the resistance to deformation increases generally, but the failure model maintained brittle fracture. After mixing the PP fiber and SF simultaneously, the stress-strain curve could be divided into three stages: the linear elastic stage, yielding stage, and failure stage. The maximum strain compared with plain cement can increase by more than 70%, and the failure model had transferred into a ductility one. This may due to the reason that the SF particles can fill the gap of cement particles and accelerate the hydration reaction, so that the internal structure of cement stone is denser. Furthermore, the strain can significantly increase with the increase of the fiber’s drag force and grip strength inside the dense structure. Figure 4 shows the failure type of specimens subjected to flexural and compressive strength tests.

#### 3.1.2. Gel Time

Table 4 shows that the PP fiber and SF possess little effect on the gel time, especially the PP fiber. For example, when w/c was 0.8, the initial and final setting time of plain cement slurry were 682 and 1293 min, respectively. For the cementitious composite at the optimal proportion, the initial and final setting time were 600 and 1185 min, respectively, which were decrease of 12% and 8%, respectively.

#### 3.1.3. Bleeding Rate

Table 4 shows that the PP fiber had a small influence on the bleeding rate. The reason may be that the PP fiber is the inert material, which cannot participate in the hydrogen reaction. Differently, SF possessed a great influence on the bleeding rate. Particularly, with the increase of SF, the bleeding rate decreased more significantly, i.e. the bleeding rate was 10.8% for plain cement at w/c = 0.8. However, that decreased to 6.67%, 2.92%, and 0%, respectively, when mixing the amount of SF = 5%, 10%, 15%. This may be due to the fact that the SF participates in the hydration reaction, and depletes the water.

Furthermore, after mixing the PP fiber and SF into the cement at the above-optimum strength proportion. The bleeding rate of the composite slurry was significantly improved compared with the plain cement slurry, especially when the w/c was relatively low. The maximum decrease rate of bleeding rate was up to 84.4%, as shown in Table 7. This indicates that the fiber-SF cementitious composite possesses favorable performance of the bleeding rate.

#### 3.1.4. Fluidity of Fiber-SF Composite Slurry at the Optimal Proportion

The slurry should possess favorable fluidity to ensure the pumpability of grouting. As shown in Table 8, the change rate of fluidity for experimental w/c was less than 30%, and the lowest fluidity of composite slurry was up to 132 mm. For the Portland cement at the w/c = 0.6 and 0.5, the fluidity was 140 and 125 mm, respectively. In addition, in practical engineering, the slurry can be pumped even when w/c = 0.4 for Portland cement. This indicates that the mixing of the presented content of SF and PP fiber displayed little impact on the slurry pumpability.

#### 3.1.5. Anti-Washout Performance of Fiber-SF Composite Slurry at the Optimal Proportion

**Figure 5:**
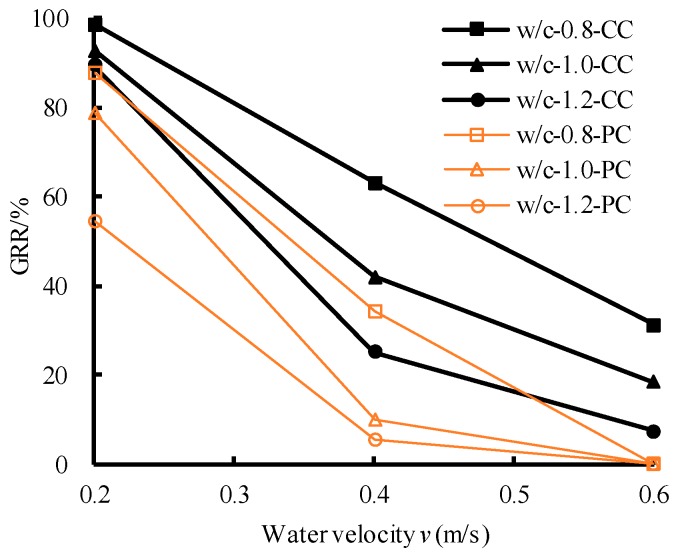
Anti-washout results between fiber-SF cementitious composite and Portland cement.

Figure 5 and Table 9 show that the plain cement slurry possessed poor anti-washout performance. When the water velocity *v* was 0.4 m/s, the *GRR* decreased sharply. While when *v* = 0.6 m/s, the *GRR* decreased to zero, indicating that the slurry was completely lost. After adding the fiber and SF, the anti-washout performance improved significantly at the low water velocity (*v* ≤ 0.4 m/s), but it slightly improved at the high velocity (*v* = 0.6 m/s). For example, when *v* = 0.4 m/s, the maximum *GRR* could be up to 63.1% (when w/c = 0.8), and the maximum increase ratio could be up to 347.8%, which was much larger than that of the plain cement slurry. However, when *v* = 0.6 m/s, the maximum *GRR* was merely 31.3%. This indicated that the composite slurry may be suitable for the low water velocity environment, but be far from the high water velocity environment. Therefore, the anti-washout performance of the slurry should be further improved.

### 3.2. Modification of PP Fiber-SF Cementitious Composite by Adding REP

**Figure 6:**
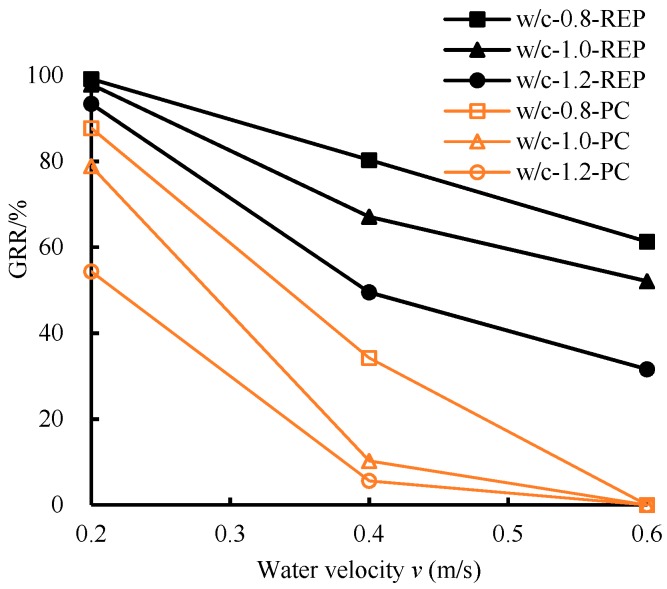
Anti-washout results between fiber-SF-REP cementitious composite and Portland cement.

To improve the anti-washout performance of the above composite slurry, REP with 3% (based on the mass of cement) was added into the composite slurry at the optimal proportion when w/c = 0.8 and 1.0, and REP with 2% was added when w/c = 1.2. The anti-washout test results of composite slurry with REP are shown in Table 9 and Figure 6.

Test results demonstrate that the *GRR* of the composite slurry was significantly improved after adding the REP. Even at the high water velocity (0.6 m/s), the *GRR* could be up to 61.3% by adjusting the w/c, which was nearly double that of the fiber-SF cement without mixing REP. In addition, with the increase of w/c, the growth rate of *GRR* increased more significantly, which could reach a maximum of 326.7%. The modification mechanism of REP may be due to the fact that the REP dispersed as a film and formed the film-forming polymer resin, which could markedly improve the cohesion strength and anti-washout properties.

Furthermore, it can be observed from Table 10 that REP could also modify the other performance, i.e., strength, bleeding rate, fluidity, and gel time. In particular, the compressive strength further increased by 30% to 10.66 MPa, while the bleeding rate was merely 1.42%. This indicates that the REP could not only improve the anti-washout properties, but could also benefit the strength, bleeding rate, and other slurry performances. In general, the emulsion with high viscosity will be formed when REP is mixed with water, causing the decrease of bleeding rate in this process and ameliorating the fluidity performance after the emulsion disperses into the slurry. In addition, the REP with the performance of a surfactant, can further increase the fluidity, workability, and homogeneity, which will cause an increase in strength. For the increase of gel time, possible reasons include the formation of emulsion that hinders the hydration reaction and the reaction of the polymer of REP and hydration products that consume the calcium hydroxide [32]. 

In summary, the above results indicated that the fiber-SF cementitious composite with REP possessed a favorable performance including strength, gel time, bleeding rate, and anti-washout properties, which may be a suitable slurry for the dynamic water environment. 

## 4. Conclusions

Considering the factors of w/c, SF content, PP fiber content, numerous experiments of strength, gel time, bleeding rate, fluidity and anti-washout properties were conducted to investigate the effects of SF and fiber on the slurry performance and the optimum strength proportion of fiber-SF slurry. On this basis, the REP polymer material was further added to improve the anti-washout properties, thereby developing a green grouting material that can restrain the hydrodynamic erosion and maintain the later strength. Through the above tests, the main conclusions are drawn as follows:

(1) A certain admixture amount of SF could significantly improve the strength and bleeding rate of the cement slurry, and the admixture of fiber could significantly modify the brittle fracture into ductile characteristics of the cement slurry.
(2) After mixing with SF and fiber simultaneously, the flexural strength and compressive strength could be significantly increased by more than 30%. In addition, the slurry fluidity could reach 132 mm, at least with the decrease of no more than 30%, which could satisfy the pumpability in the practical engineering.
(3) When the flow velocity was relatively small (*v* ≤ 0.4 m/s), the anti-washout properties could be significantly increased by mixing the fiber and SF and the maximum *GRR* could be up to 98.7%. However, the maximum *GRR* at the high flow velocity (*v* = 0.6m/s) was a mere 31.3%. After adding the REP material, the anti-washout property of the composite slurry was further improved, especially in the high flow velocity environment, where the maximum *GRR* at *v* = 0.6m/s could be up to more than 60%. Simultaneously, the admixture of REP could further increase the other performance such as strength, fluidity, and bleeding rate. The developed composite slurry possessed favorable performance in the dynamic water environment, which can be used as a reference for the engineers.

## Figures and Tables

**Table 1:** Chemical composition of cement and SF (mass %).

Type	SiO_2_	AL_2_O_3_	Fe_2_O_3_	Ca O + MgO_2_	K_2_O + Na_2_O	Loss	Water
Cement	21.28	5.32	3.37	70.15	-	0.74	-
SF	≥90	≤1.5	≤2	≤2	≤2	-	≤5

**Table 2:** Performance index of PP fiber.

Density (g·cm^−3^)	Diameter (μm)	Length (mm)	Fracture Strength (MPa)	Fracture Elongation (%)	Young‘s Modulus (GPa)	Melting Point (°C)
0.91	31	9	≥400	≤2	≥3.5	160

**Table 3:** Performance index of REP.

Bulk Density (g·cm^−3^)	Diameter (μm)	Viscosity of 50% Aqueous Solution (mm)	Solid Content (%)	Ash Content (1000 °C) (%)
0.4–0.6	80	≥10	98	15 ± 2

**Table 4:** Strength and gel time results for the fiber-silica fume composite slurry.

w/c	PP (%)	SF (%)	Compressive Strength (MPa)	Flexural Strength (MPa)	Bleeding Rate (%)	Gel Time (min)	
						Initial Setting	Final Setting
0.8	0.0	0	6.04	1.67	10.80	682	1293
	0.0	5	6.26	1.78	6.67	630	1212
	0.0	10	6.91	1.89	2.92	605	1185
	0.0	15	6.56	1.51	0.00	600	1170
	0.1	0	6.11	1.68	10.50	683	1295
	0.1	5	6.14	1.68	6.45	635	1212
	0.1	10	7.63	1.98	2.63	605	1185
	0.1	15	7.12	1.87	0.00	600	1170
	0.3	0	6.18	1.72	10.16	683	1295
	0.3	5	6.22	1.78	5.87	635	1215
	0.3	10	7.88	2.12	2.43	605	1185
	0.3	15	7.26	1.98	0.00	600	1170
	0.5	0	6.26	1.81	10.02	685	1295
	0.5	5	6.31	1.88	5.65	635	1210
	0.5	10	8.03	2.23	1.68	600	1185
	0.5	15	7.38	2.08	0.00	600	1180
1.0	0.0	0	4.84	1.45	20.40	713	1320
	0.0	5	5.56	1.62	11.67	695	1300
	0.0	10	4.76	1.39	5.00	655	1260
	0.0	15	4.79	1.06	2.92	625	1230
	0.1	0	5.08	1.53	18.58	713	1320
	0.1	5	5.92	1.59	10.23	685	1300
	0.1	10	5.41	1.24	4.87	660	1260
	0.1	15	5.09	1.15	2.79	655	1230
	0.3	0	5.25	1.59	18.33	713	1320
	0.3	5	6.03	1.61	10.10	685	1295
	0.3	10	5.52	1.28	4.65	665	1255
	0.3	15	4.95	1.08	2.67	655	1255
	0.5	0	5.31	1.61	18.92	712	1320
	0.5	5	6.07	1.68	5.12	685	1295
	0.5	10	5.62	1.35	4.21	660	1255
	0.5	15	4.82	0.99	2.73	650	1255
1.2	0.0	0	4.06	1.05	31.25	745	1360
	0.0	5	4.21	1.11	25.83	715	1330
	0.0	10	3.67	0.55	17.50	680	1305
	0.0	15	2.56	0.46	5.00	660	1290
	0.1	0	4.45	1.09	30.83	745	1360
	0.1	5	4.74	1.16	25	715	1330
	0.1	10	3.88	0.59	16.70	680	1305
	0.1	15	2.64	0.48	4.63	660	1290
	0.3	0	4.21	1.06	29.92	745	1360
	0.3	5	4.66	1.13	23.83	715	1330
	0.3	10	3.82	0.55	15.84	680	1305
	0.3	15	2.52	0.42	4.22	660	1290
	0.5	0	4.13	0.61	29.58	745	1360
	0.5	5	4.57	1.11	22.76	715	1330
	0.5	10	3.75	0.45	14.35	680	1305
	0.5	15	2.38	0.35	3.43	660	1290

**Table 5:** Tests of Between-Subjects Effects for Compressive strength.

Source	Type III Sum of Squares	df	Mean Square	F	Sig.
Corrected Model	77.729 ^a^	8	9.716	26.750	0.000
Intercept	1339.431	1	1339.431	3687.640	0.000
wc	72.143	2	36.072	99.311	0.000
PP	1.124	3	0.375	1.032	0.389
SF	4.461	3	1.487	4.094	0.013
Error	14.166	39	0.363		
Total	1431.325	48			
Corrected Total	91.894	47			

^a^. R Squared = 0.846 (Adjusted R Squared = 0.814).

**Table 6:** Strength results of composite slurry and plain Portland cement slurry.

w/c	Strength of Portland Cement (MPa)		Strength of Composite Slurry (MPa)		Strength Increase Rate (%)	
	Compressive Strength	Flexural Strength	Compressive Strength	Flexural Strength	Compressive Strength	Flexural Strength
0.8	6.04	1.67	8.03	2.23	32.9	33.5
1.0	4.84	1.45	6.07	1.68	25.4	15.9
1.2	4.06	1.05	4.74	1.16	16.7	10.5

**Table 7:** Comparison results of bleeding rate between composite slurry and Portland cement.

w/c	Bleeding Rate of the Portland Cement (%)	Bleeding Rate of the Composite Slurry (%)	Change Rate of Bleeding (%)
0.8	10.80	1.68	84.40
1.0	20.40	7.88	61.37
1.2	31.25	25.00	20.00

**Table 8:** Comparison results of fluidity between composite slurry and Portland cement.

w/c	Fluidity of Portland Cement (mm)	Fluidity of Composite Slurry (mm)	Change Rate of Fluidity (%)
0.8	180	132	26.7
1.0	340	265	22.1
1.2	400	335	16.3

**Table 9:** Anti-washout results.

w/c	*v* (m/s)	GRR (%)			Growth Rate of GRR (%)	
		Plain Cement Slurry	Fiber-SF Slurry	Fiber-SF-REP Slurry	Fiber-SF Slurry Compared to Portland Cement	Fiber-SF-REP Slurry Compared to Fiber-SF Slurry
0.8	0.2	87.7	98.7	99.2	12.5	0.5
0.8	0.4	34.3	63.1	80.3	84.3	27.2
0.8	0.6	0.0	31.3	61.3	/	96.2
1.0	0.2	78.9	92.8	97.9	17.7	5.5
1.0	0.4	10.3	42.0	67.1	309.4	59.9
1.0	0.6	0.0	18.6	52.1	/	180.1
1.2	0.2	54.4	89.4	93.4	64.4	4.5
1.2	0.4	5.6	25.1	49.5	347.8	96.9
1.2	0.6	0.0	7.4	31.6	/	326.7

**Table 10:** Test results after adding the REP into the fiber-SF cementitious composite.

w/c	Strength (MPa)		Bleeding Rate (%)	Fluidity (mm)	Gel Time (min)	
	Compressive Strength	Flexural Strength			Initial Setting	Final Setting
0.8	10.66	2.32	1.42	139	645	1230
1.0	8.25	1.78	4.26	287	715	1325
1.2	6.34	1.37	21.3	372	735	1365
